# Rheology of Emulsions Thickened by Starch Nanoparticles

**DOI:** 10.3390/nano12142391

**Published:** 2022-07-13

**Authors:** Anuva Pal, Rajinder Pal

**Affiliations:** Department of Chemical Engineering, University of Waterloo, Waterloo, ON N2L 3G1, Canada; a26pal@uwaterloo.ca

**Keywords:** emulsion, nanoparticles, starch, rheology, viscosity, non-Newtonian, shear-thinning

## Abstract

The rheology of oil-in-water (O/W) emulsions thickened by starch nanoparticles is investigated here. The starch nanoparticle concentration is varied from 0 to 25 wt% based on the matrix aqueous phase. The oil concentration is varied from 0 to 65 wt%. At a given nanoparticle concentration, the emulsions are generally Newtonian at low oil concentrations. The emulsions become shear-thinning at high oil concentrations. The increase in nanoparticle concentration at a given oil concentration increases the consistency of the emulsion and enhances the shear-thinning behavior of emulsion. The rheological behavior of emulsions is described reasonably well by a power-law model. The consistency index of the emulsion increases with the increases in nanoparticle and oil concentrations. The flow behavior index of emulsion decreases with the increases in nanoparticle and oil concentrations, indicating an increase in the degree of shear-thinning in emulsion.

## 1. Introduction

Emulsions are dispersions of two immiscible liquids such as oil and water. In the absence of any stabilizer additive such as a surfactant, the emulsions are unstable and readily separate into oil and water layers when left unstirred. To stabilize the emulsions, a surface-active agent (surfactant) is added. The surfactant serves two functions: (1) it decreases the interfacial tension between oil and water, thereby making the formation of emulsion easier, and (2) it stabilizes the droplets against coalescence [1,2]. 

Many natural and processed food products are emulsions. Examples of food emulsions include milk, mayonnaise, butter, margarine, and salad dressings [2,3,4,5,6,7]. Table 1 gives a brief description of the compositions of some emulsion-based food products. Emulsions also find applications in many other industries including petroleum, pharmaceuticals, cosmetics, agriculture, explosives, paints, and lubricants [2,4,6,7,8,9,10].

One problem with emulsions is that they undergo creaming and sedimentation effects under the influence of gravity due to differences in the densities of the oil and water phases [11]. Creaming occurs in oil-in-water (O/W) emulsions where light oil droplets tend to rise through the heavy aqueous phase (matrix fluid), whereas sedimentation occurs in water-in-oil (W/O) emulsions, where heavy water droplets tend to fall through the light oil phase. Sedimentation and creaming in emulsions are undesirable as they affect the shelf-life of the product [12]. To minimize creaming and sedimentation in emulsions, rheology modifiers (thickeners) are usually added to the matrix phase. According to the Stokes law, the creaming velocity of oil droplets in O/W emulsions or the sedimentation velocity of water droplets in W/O emulsions is inversely related to the viscosity of the matrix fluid (continuous phase). Thus, the creaming and sedimentation of droplets can be minimized by increasing the viscosity of the matrix fluid.

One commonly used approach to increase the viscosity of the matrix phase of emulsions is to incorporate a polymeric additive in the matrix fluid [13,14]. Alternatively, one can use nanoparticles to thicken the matrix fluid of emulsions. While a few studies have been published on the rheology of polymer-thickened emulsions [13,14,15,16,17,18,19], little or no work has been reported on nanoparticle-thickened emulsions. However, it should be noted that many studies have been published in recent years on the adsorption of nanoparticles at the oil–water interface to stabilize the droplets against coalescence. Such nanoparticle-stabilized emulsions are referred to as Pickering emulsions [20,21,22,23,24,25,26,27,28]. In several published studies on Pickering emulsions [29,30,31,32,33], the nanoparticles used to stabilize the droplets are produced from native and modified starches. The usage of starches to produce nanoparticles has the advantage that they are renewable, biocompatible, biodegradable, and affordable.

**Figure 1:**
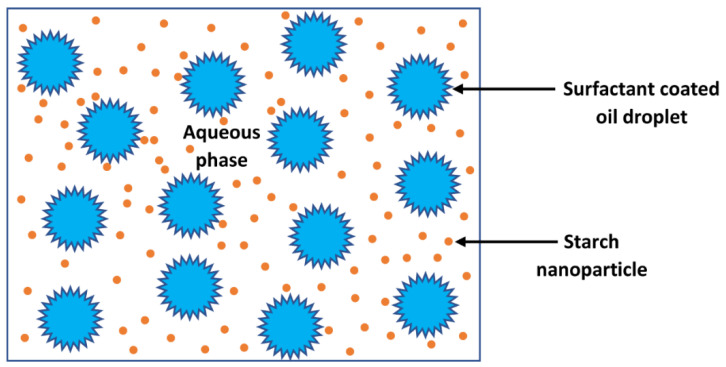
Schematic of the type of emulsions investigated in the present work.

In this article, we report new results on the rheology of nanoparticle-thickened O/W emulsions over a broad range of nanoparticle and oil concentrations. The nanoparticle concentration was varied from 0 to approximately 25 wt% based on the matrix fluid (aqueous phase). The oil concentration of emulsion was varied from 0 to approximately 65 wt%. The emulsions were highly stable with respect to coalescence due to the presence of surfactant at the surface of the droplets. Figure 1 shows a schematic representation of the type of emulsions investigated. 

## 2. Experimental Work

### 2.1. Materials

**Figure 2:**
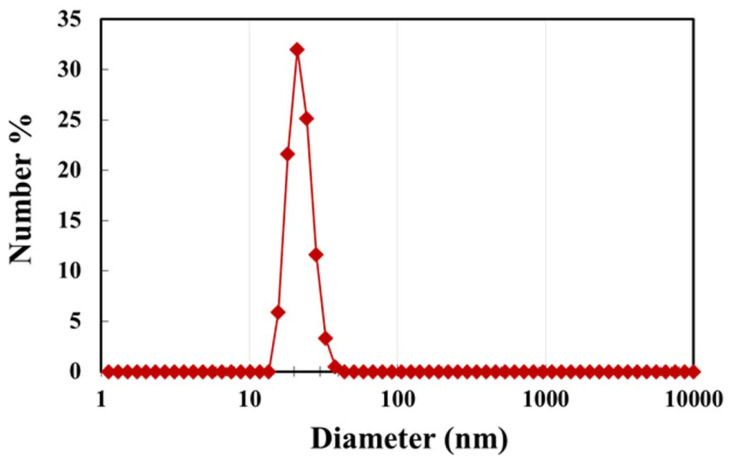
Size distribution of starch nanoparticles [34].

The emulsions of oil-in-water (O/W) type were prepared using the following materials: deionized water, food grade white mineral oil, nanoparticles, and non-ionic water-soluble surfactant. The oil used was Petro-Canada white mineral oil (code: Purity FG WO-15), supplied by Boucher and Jones Fuels, Waterloo (ON, Canada). The viscosity of the batch of oil used in this study was 27.62 mPa.s at 21 °C. The nanoparticles used were experimental grade starch biopolymer nanoparticles supplied by EcoSynthetix Inc. (Burlington, ON, Canada). They are manufactured through reactive extrusion by modifying native starch. The mean diameter of the starch nanoparticles was approximately 21 nm (see Figure 2 for particle size distribution reported in our earlier study [34]). The surfactant used was Triton X-100, a commercially available non-ionic surfactant supplied by Union Carbide.

### 2.2. Preparation of Nanoparticle Suspensions

**Figure 3:**
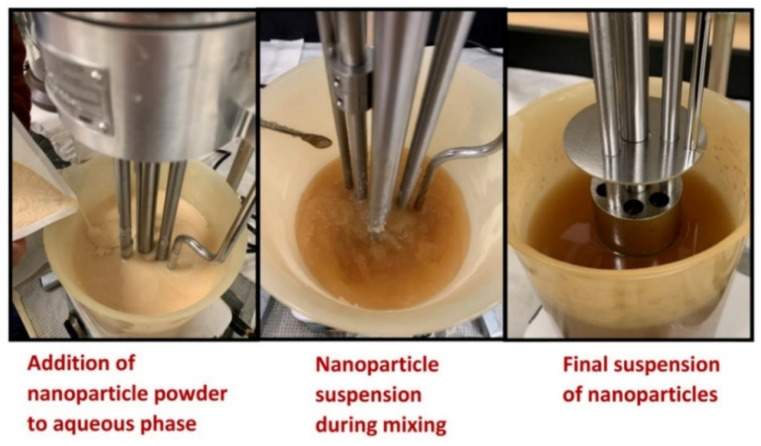
Preparation of suspension of starch nanoparticles.

The nanoparticle suspension was prepared at room temperature (≈21 °C) by slowly adding a known amount of starch nanoparticles into a known amount of aqueous phase while maintaining mixing of the suspension using a variable speed homogenizer (Gifford-Wood, model 1 L, NOV process and flow technologies, Dayton, OH, USA). The aqueous phase of the suspension consisted of deionized water containing ≈1 wt% of surfactant Triton X-100 and ≈0.15 wt% biocide (Thor Acticide GA). The biocide was added to prevent any bacterial growth in the starch suspension. The suspension was agitated in the homogenizer at high speed for at least 30 min until the starch powder was fully dispersed. The nanoparticle suspension was left overnight to remove any air entrapped during the homogenization process. Six suspensions of different nanoparticle concentrations ranging from 0 to approximately 25 wt% were prepared. The nanoparticle concentration of the suspension was increased in increments of approximately 5 wt%. Figure 3 shows the preparation process for the suspension of starch nanoparticles.

### 2.3. Preparation of O/W Emulsions

The emulsions of oil-in-water (O/W) type were prepared by slowly adding a known amount of oil to a known amount of starch nanosuspension while maintaining mixing of the fluids using a homogenizer. After addition of the required amount of oil, the fluids were sheared in the homogenizer at high speed for at least 30 min. The emulsion thus prepared was left overnight to remove any air entrapped during the homogenization process. A higher oil concentration emulsion was prepared by slowly adding a known amount of oil to an existing lower concentration emulsion while mixing the fluids in the homogenizer. After the addition of oil, the fluids were sheared in the homogenizer at high speed for more than 30 min. The oil concentration of the emulsion was generally increased in increments of approximately 10 wt%. The range of oil concentration for a given nanoparticle suspension was varied from 0 to approximately 65 wt%. 

Table 2 gives complete information about the compositions of the emulsions investigated in this study. Note that the matrix phase of the emulsions consisted of suspension of starch nanoparticles in aqueous phase. The aqueous phase itself consisted of ≈1 wt% surfactant Triton X-100 and ≈0.15 wt% of biocide. 

### 2.4. Measurements

The rheological measurements were carried out using two co-axial cylinder devices, namely: Fann viscometer and Haake viscometer. The relevant dimensions of the devices are given in Table 3. In the Fann viscometer, the inner cylinder is stationary, and the outer cylinder rotates. There are 12 speeds ranging from 0.9 to 600 rpm. In the Haake viscometer, the inner cylinder rotates, and the outer cylinder is stationary. There are 30 speeds ranging from 0.01 to 512 rpm. The viscometers were calibrated using standards of known viscosities. The viscosity measurements were carried out at room temperature (≈21 °C). 

The droplets of emulsions were observed using a Zeiss optical microscope with transmitted light. The photomicrographs of emulsion droplets were taken using a camera. The emulsion samples were diluted with the surfactant solution (≈1 wt%) before observation under the microscope. The dilution of the emulsion sample was necessary to allow the transmitted light to pass through the sample. The emulsion sample was diluted with the dispersion medium (≈1 wt% surfactant solution) of the emulsion to avoid any destabilization and coalescence of droplets. This technique of observing the droplet sizes microscopically by dilution of the emulsion sample with the same continuous-phase fluid as that of the emulsion has been used extensively in the literature [35,36,37].

## 3. Results and Discussion

### 3.1. Rheology of Nanoparticle Suspensions

**Figure 4:**
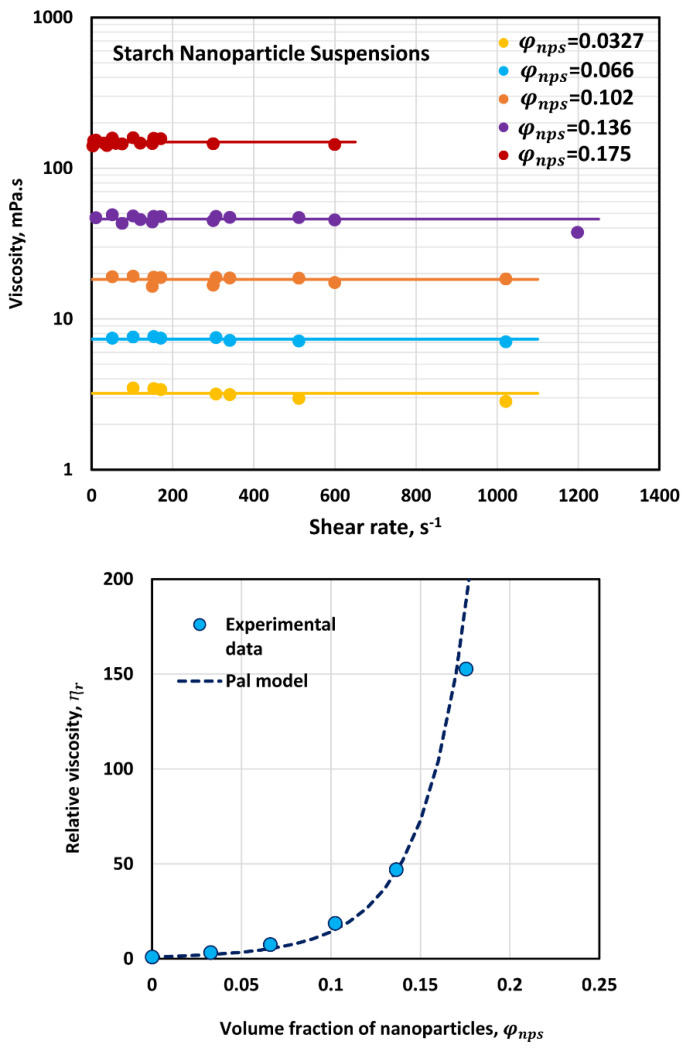
Viscous behavior of suspensions of starch nanoparticles.

Figure 4 shows the flow behavior of suspensions of starch nanoparticles. The nanoparticle suspensions are Newtonian in that the viscosity is constant independent of the shear rate. The viscosity increases with the increase in nanoparticle concentration. The relative viscosity of nanoparticle suspensions can be described adequately by the following Pal viscosity model [38] for suspensions:

(1) $${ⴄ}_{r}={\left[1-\left\{1+\left(\frac{1-\varphi_{m}}{\varphi_{m}^{2}}\right)\right\}\varphi \right]}^{-\left[ⴄ\right]}$$

where ${ⴄ}_{r}$ is the relative viscosity, φ is the volume fraction of particles, $\varphi_{m}$ is the maximum packing volume fraction of particles, and [ⴄ] is the intrinsic viscosity of suspension. The value of $\varphi_{m}$ is taken as 0.637, corresponding to random packing of spheres. As can be seen in Figure 4, Equation (1) fits the data very well with [ⴄ]=21.28. The intrinsic viscosity for rigid spherical particles is 2.5. A high value of [ⴄ] observed for starch nanoparticles is indicative of the swelling of nanoparticles. It is well known that starch nanoparticles undergo significant swelling when dispersed in aqueous phase [39].

### 3.2. Rheology of O/W Emulsions without Nanoparticles

**Figure 5:**
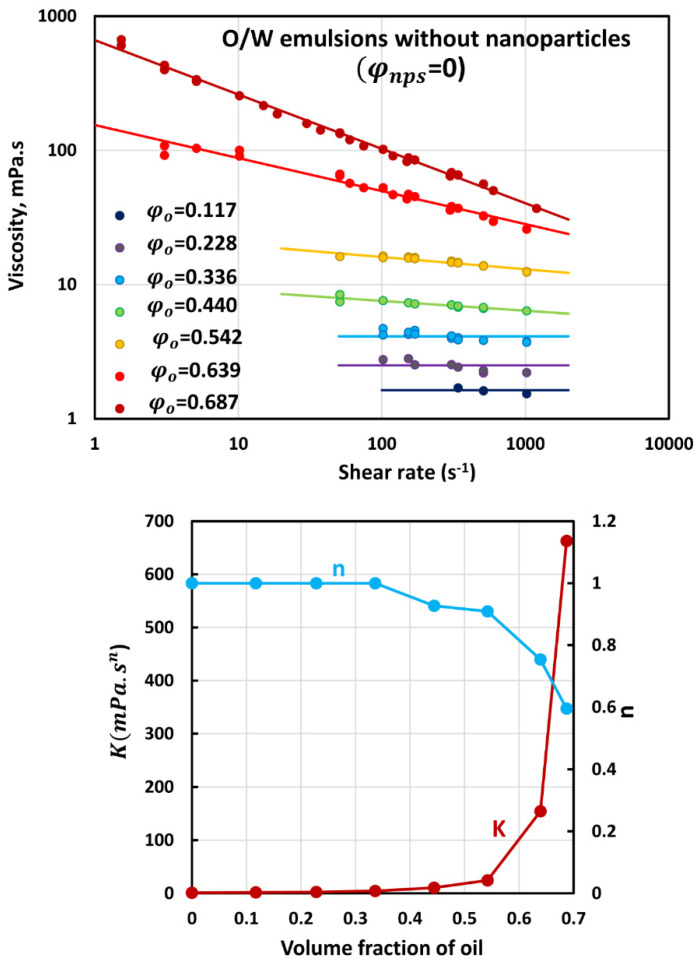
Viscous behavior of O/W emulsions without nanoparticles.

Figure 5 shows the flow behavior of O/W emulsions without nanoparticles. The data are shown for different oil concentrations. The emulsions are Newtonian up to oil volume fraction of 0.336. At higher oil volume fractions, the emulsions become shear-thinning in that the viscosity decreases with the increase in shear rate. The viscosity versus shear rate data can be described using the power-law model:

(2) $$\tau =K{\dot{\gamma }}^{n}$$

where τ is the shear stress, $\dot{\gamma }$ is the shear rate, K is the consistency index, and n is the flow-behavior index. The flow behavior index n decreases whereas the consistency index increases with the increase in oil volume fraction. Thus, emulsions become more shear-thinning and viscous with the increase in oil volume fraction.

It is worth mentioning that O/W emulsions tend to develop yield-stress and become viscoelastic when the oil droplet concentration becomes significantly larger than the maximum packing volume fraction of droplets ($\varphi_{o}>0.75$). Such emulsions are referred to as HIPREs (high internal phase ratio emulsions) or gel emulsions. The rheological behavior of gel emulsions is governed by a network of interfacial films [40,41]. In the present work, the emulsions investigated were not HIPREs or gel emulsions as the droplet concentration was less than approximately 70 percent by volume. 

### 3.3. Rheology of O/W Emulsions Thickened by Starch Nanoparticles

**Figure 6:**
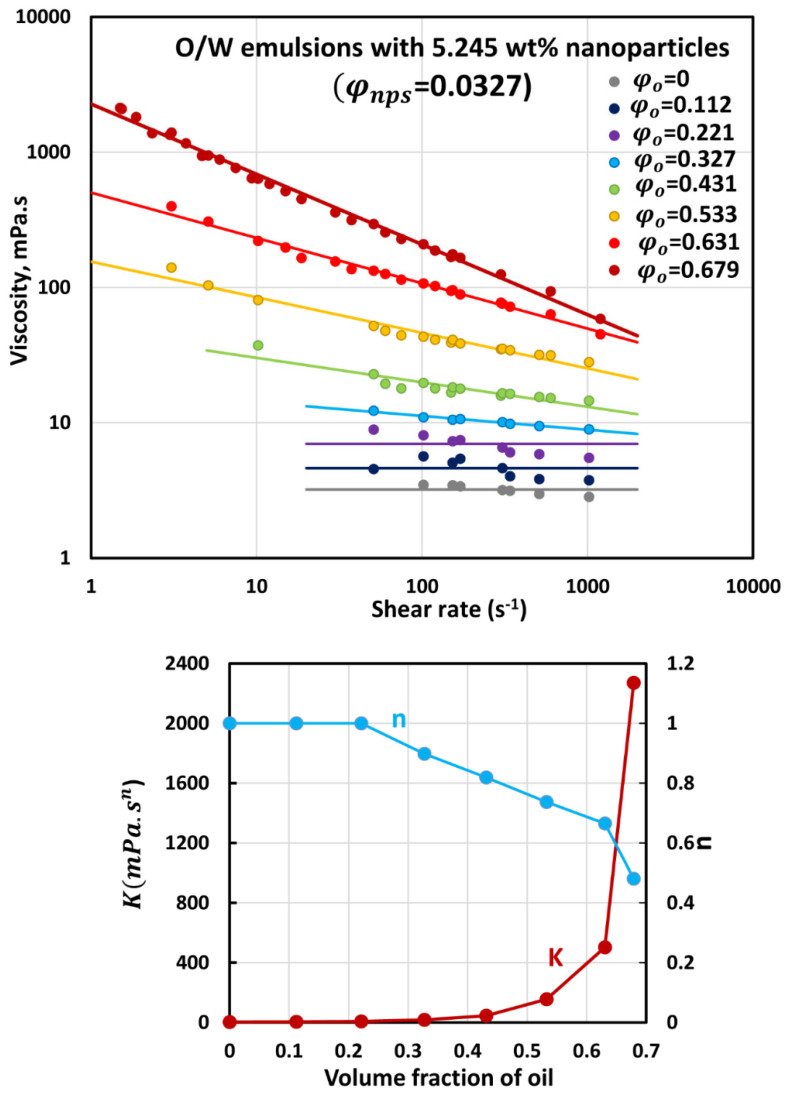
Flow behavior of O/W emulsions thickened with starch nanoparticles at nanoparticle concentration of 5.245 wt% ($\varphi_{nps}=0.0327$).

**Figure 7:**
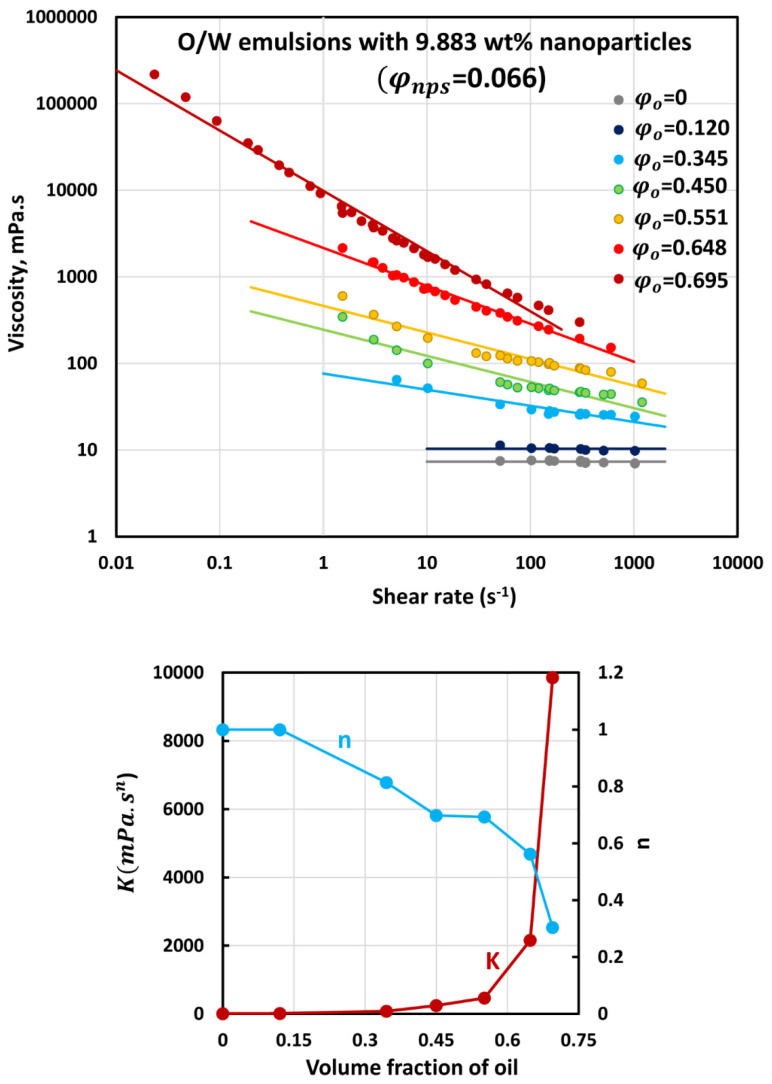
Flow behavior of O/W emulsions thickened with starch nanoparticles at nanoparticle concentration of 9.883 wt% ($\varphi_{nps}=0.066$).

**Figure 8:**
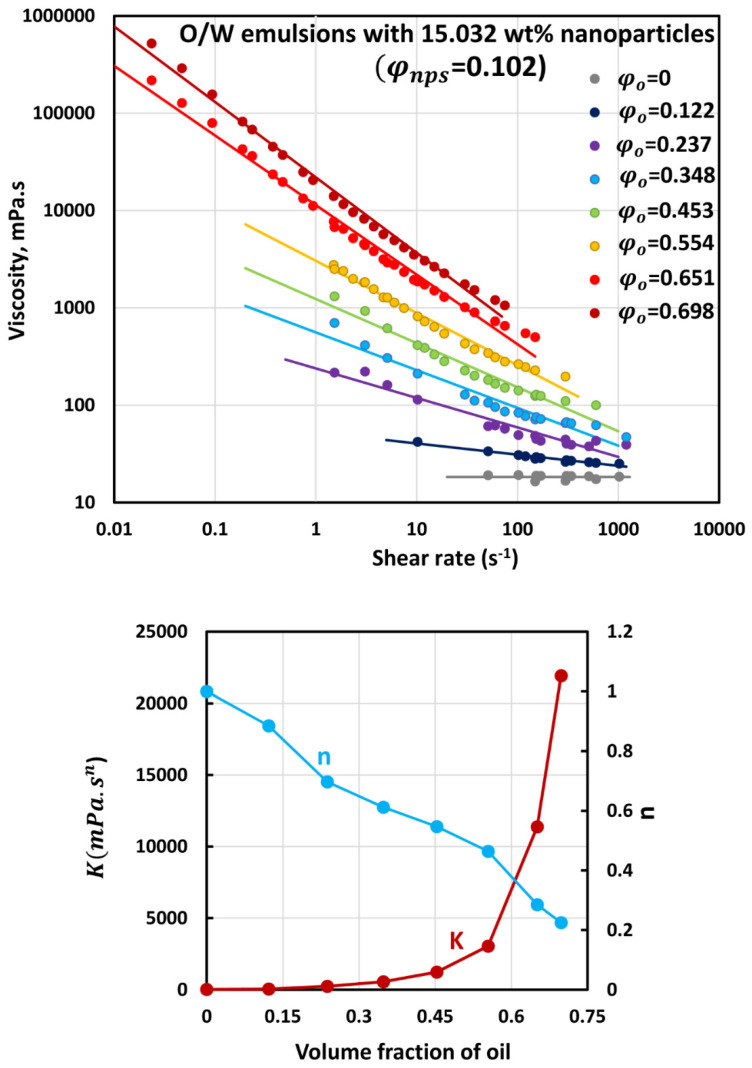
Flow behavior of O/W emulsions thickened with starch nanoparticles at nanoparticle concentration of 15.032 wt% ($\varphi_{nps}=0.102$).

**Figure 9:**
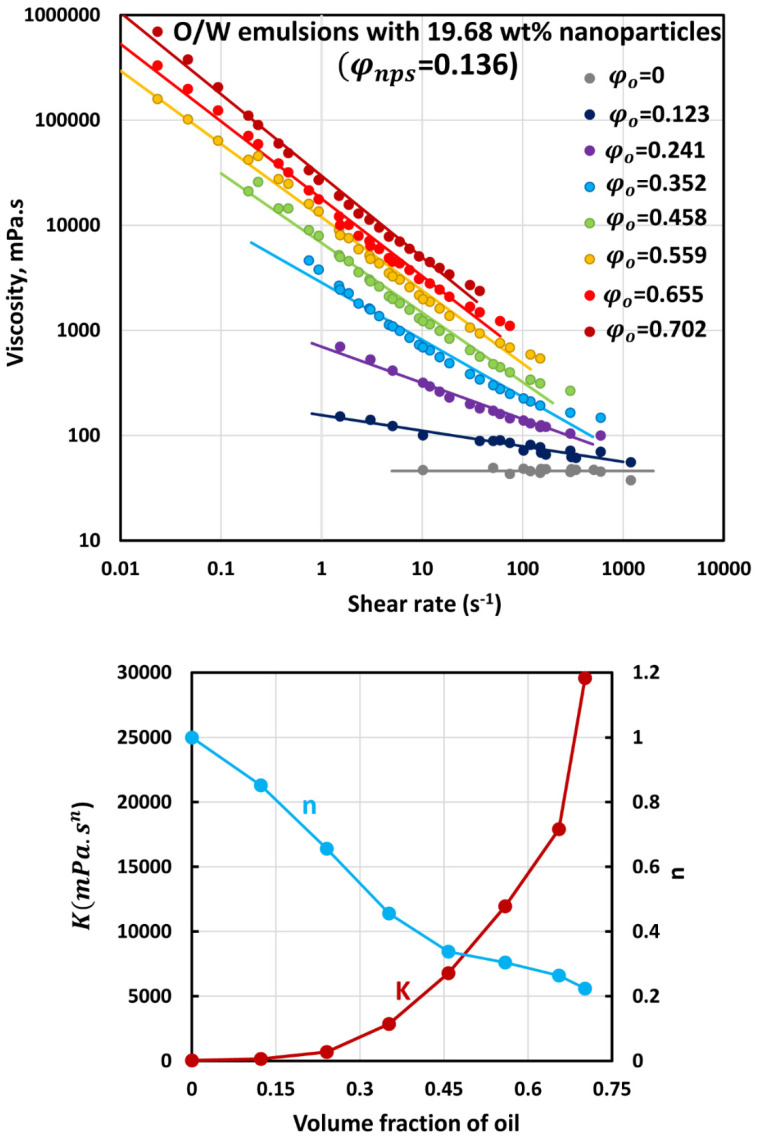
Flow behavior of O/W emulsions thickened with starch nanoparticles at nanoparticle concentration of 19.68 wt% ($\varphi_{nps}=0.136$).

**Figure 10:**
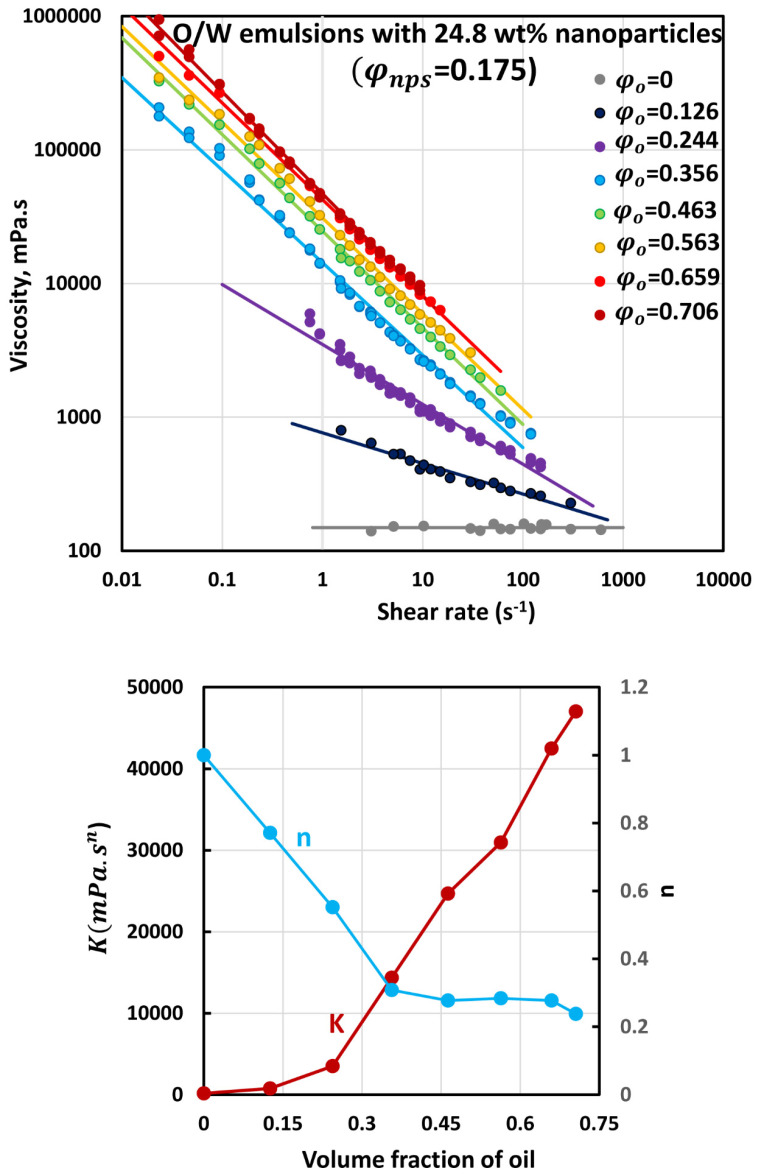
Flow behavior of O/W emulsions thickened with starch nanoparticles at nanoparticle concentration of 24.8 wt% ($\varphi_{nps}=0.175$).

The flow curves (viscosity versus shear rate plots) of O/W emulsions thickened with different concentrations of starch nanoparticles are shown in Figure 6, Figure 7, Figure 8, Figure 9 and Figure 10. The flow curves for nanoparticle-thickened emulsions can be described reasonably well using the power-law model, Equation (2). For any given nanoparticle concentration, the flow curve becomes steeper with the increase in oil concentration. Thus, the degree of shear-thinning in emulsion increases with the increase in oil concentration while the flow behavior index n decreases. However, the consistency of the emulsion, as reflected in the consistency index K, increases with the increase in oil concentration at any given nanoparticle concentration. 

**Figure 11:**
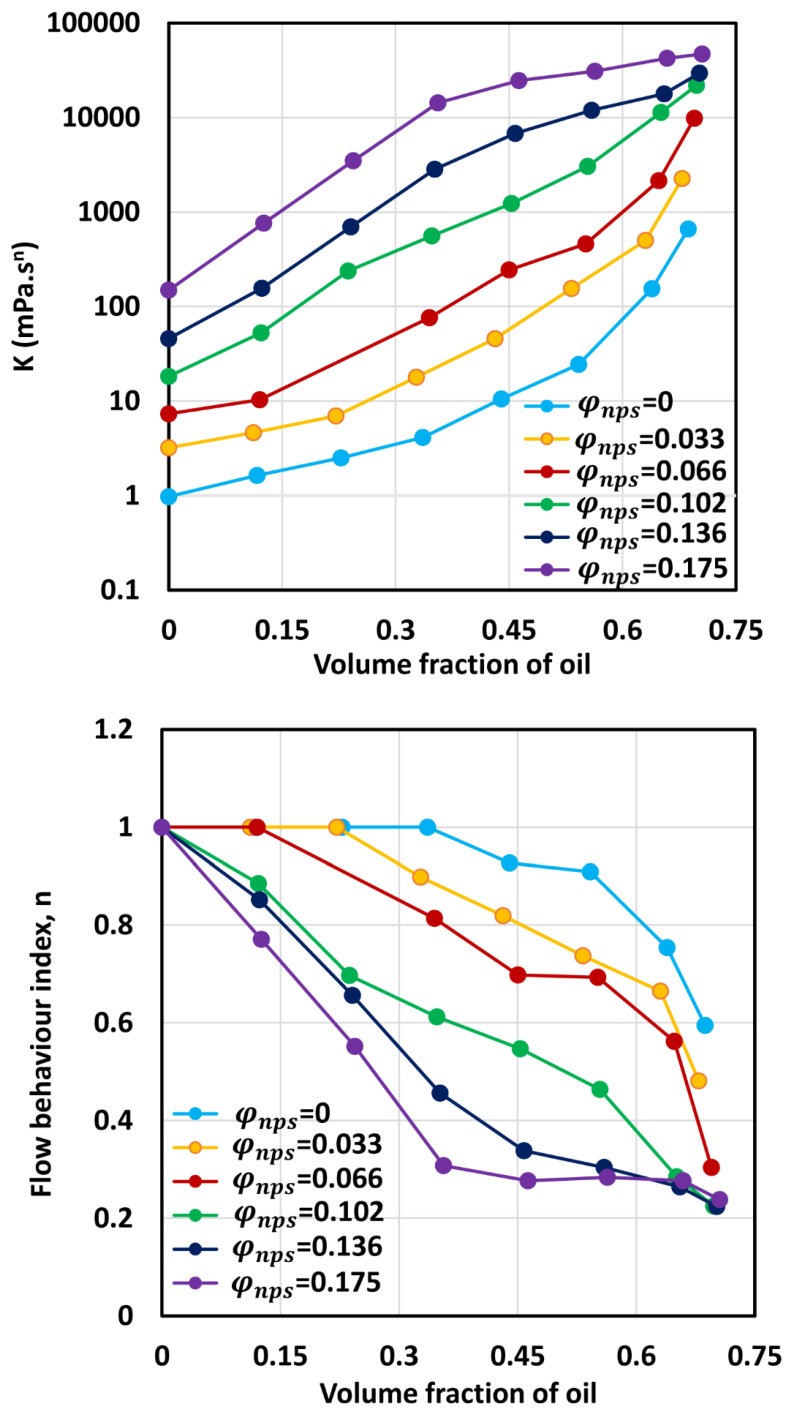
Consistency index (K) and flow behavior index (n) of O/W emulsions thickened with starch nanoparticles.

Figure 11 compares the consistency index K and flow behavior index n values for O/W emulsions thickened with different concentrations of nanoparticles. For any given nanoparticle concentration, the consistency index increases, and the flow behavior index decreases with the increase in oil concentration, as noted earlier. Interestingly, at any given volume fraction of oil, the consistency index increases, whereas the flow behavior index decreases with the increase nanoparticle concentration of the matrix fluid (aqueous phase).

**Figure 12:**
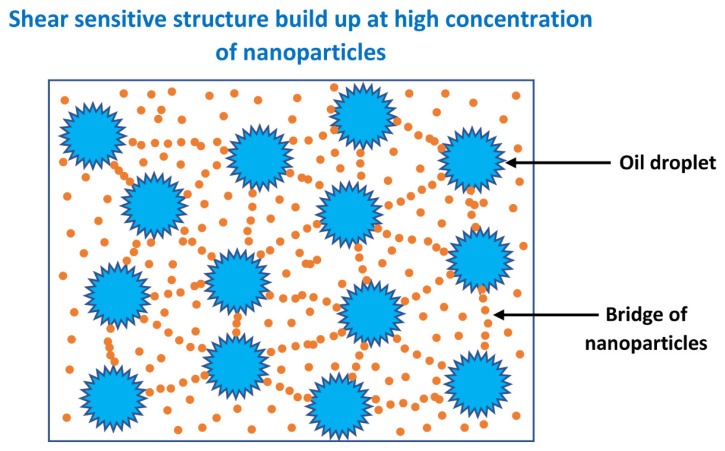
Shear-sensitive structure build-up in emulsions at high nanoparticle concentrations.

**Figure 13:**
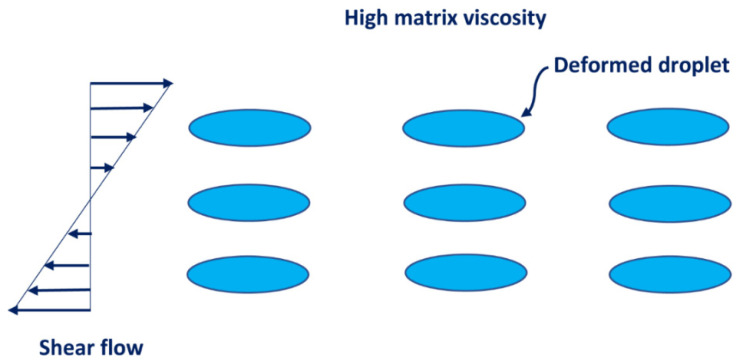
Large deformation of droplets at high concentration of nanoparticles at any given shear rate.

**Figure 14:**
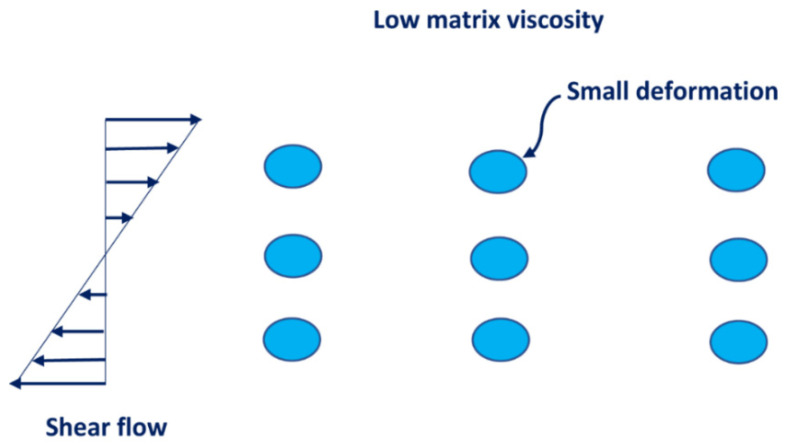
Small deformation of droplets at low concentration of nanoparticles at any given shear rate.

The increase in consistency of an emulsion with the increase in nanoparticle concentration is expected as the consistency of emulsion is directly proportional to the consistency of matrix fluid of the emulsion which increases with the increase in nanoparticle concentration. The increase in the degree of shear-thinning (decrease in flow behavior index n) with the increase in nanoparticle concentration at a given oil concentration could be explained in terms of structure build-up [42] and the deformation of droplets [7]. With the increase in nanoparticle concentration, the nanoparticles likely form bridges between the neighboring oil droplets, as shown in Figure 12. This structure is more sensitive to shear and hence the emulsion exhibits a greater degree of shear-thinning. The other mechanism for the enhancement of the shear-thinning of emulsions is the deformation of droplets under shear. At high concentration of nanoparticles, the matrix viscosity is high and therefore the droplets undergo a higher degree of deformation at a given shear rate (see Figure 13). At a low concentration of nanoparticles, the matrix viscosity is low, and the droplets deform only marginally at the same shear rate (see Figure 14). Consequently, for the same change in shear rate, emulsions with a high concentration of nanoparticles experience a greater decrease in viscosity as compared with emulsions at a lower concentration of nanoparticles. 

### 3.4. Droplet Size of Emulsions

**Figure 15:**
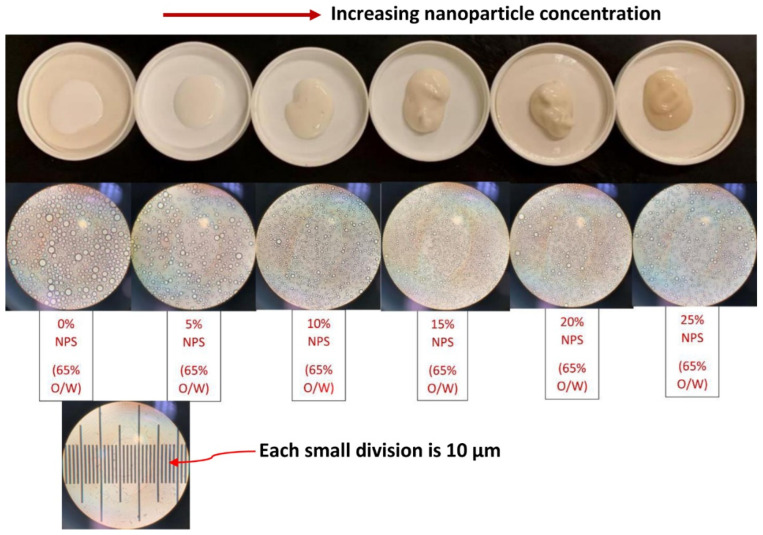
Images of emulsion samples and their corresponding droplets with increasing nanoparticle concentration (wt% NPS) at a fixed oil concentration of approximately 65 wt%.

Figure 15 shows the images of emulsion samples and their corresponding droplets with increasing nanoparticle concentration at a fixed oil concentration of approximately 65 wt%. With the increase in nanoparticle concentration, the consistency of emulsion changes from fluid-like material to paste. The droplet size also generally decreases with the increase in nanoparticle concentration. Table 4 summarizes the Sauter mean diameters of emulsions. 

**Figure 16:**
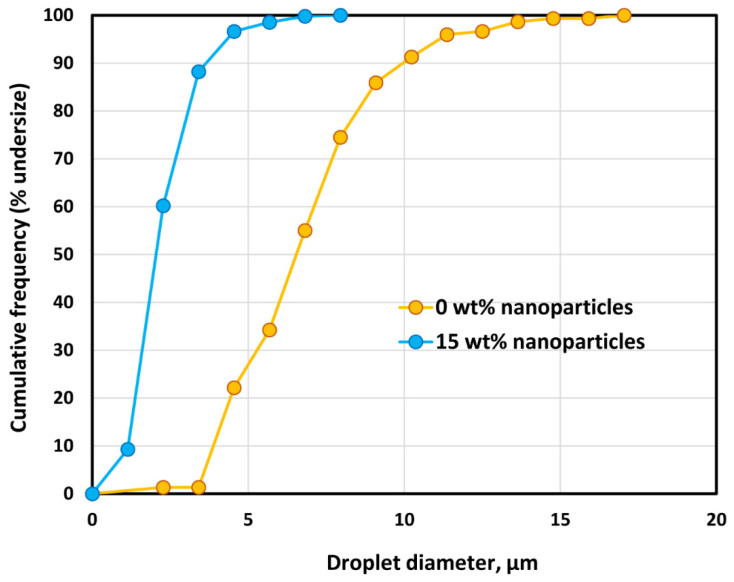
Droplet size distributions of O/W emulsions without and with nanoparticles. The oil concentration of emulsions is approximately 65 wt%.

The Sauter mean diameter decreases from 9 µm to about 4–5 µm when the nanoparticle concentration is increased from 0 to 25 wt% at a fixed oil concentration of approximately 65 wt%. It should also be noted that the droplet size distribution of emulsions is affected by the nanoparticle concentration. As an example, Figure 16 compares the droplet size distributions of O/W emulsions without and with nanoparticles. The oil concentration is approximately 65 wt% and the nanoparticle concentration is 15 wt%. The O/W emulsion thickened by nanoparticles has a narrower size distribution of droplets. 

It is well known that a decrease in the droplet size of emulsion increases its consistency [35,36,37]. Thus, the change in consistency of emulsion from fluid to paste-like material with the increase in nanoparticle concentration (see Figure 15) is partly due to a decrease in average droplet size and partly due to increase in the viscosity of the matrix phase. 

**Figure 17:**
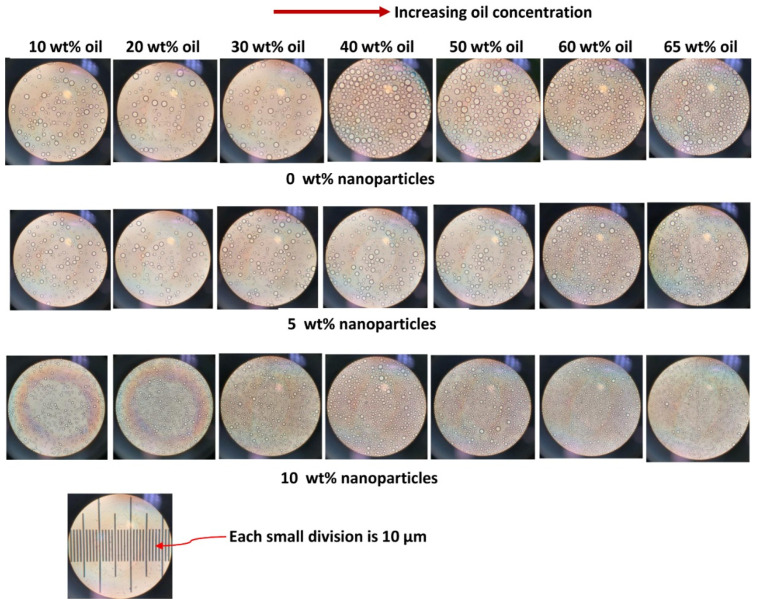
Images of emulsion droplets with increasing oil concentration at different nanoparticle concentrations.

The effect of increasing oil concentration on the droplet sizes while keeping the nanoparticle concentration fixed is shown in Figure 17. The droplet sizes do not vary significantly with the increase in oil concentration. 

### 3.5. Stability of Emulsions

**Figure 18:**
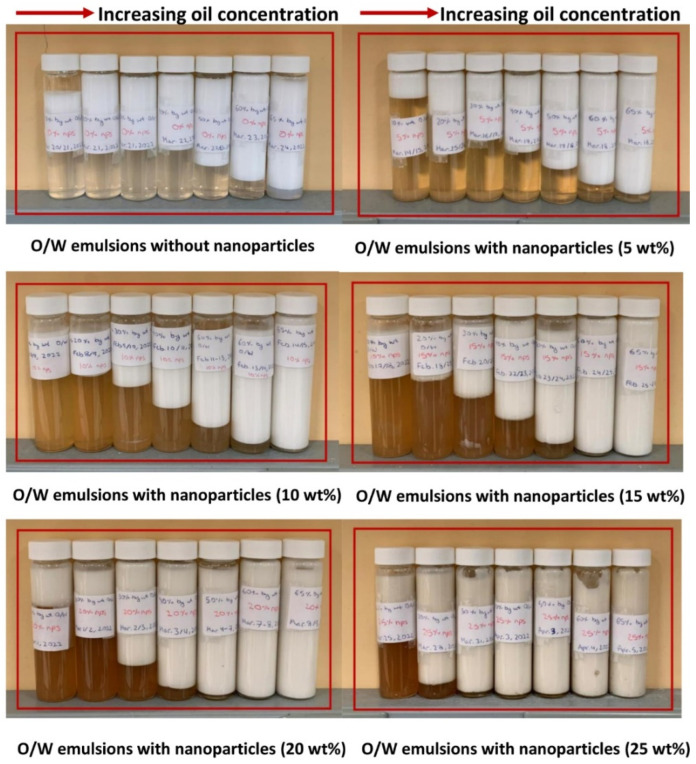
Creaming in O/W emulsions when left unstirred for several weeks.

**Figure 19:**
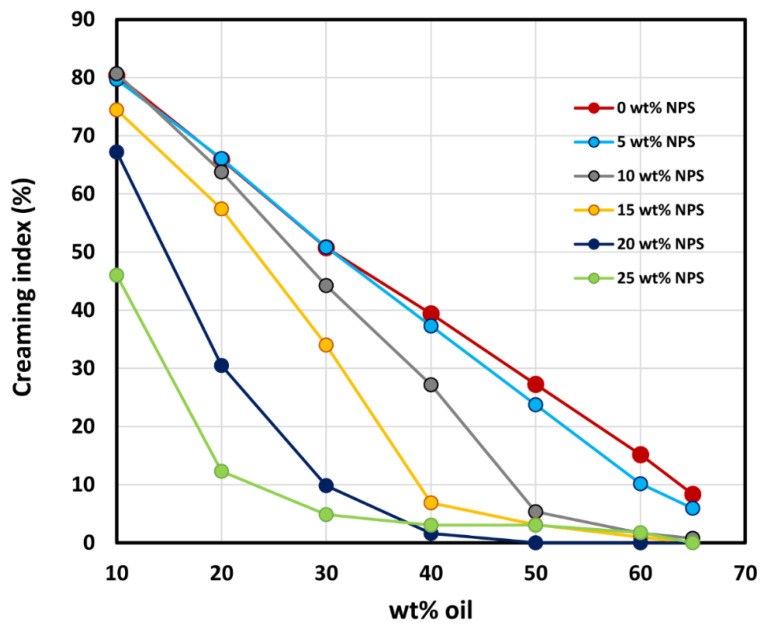
Percent creaming index of O/W emulsions when left unstirred for several weeks.

The emulsions produced were highly stable with respect to coalescence. No deterioration of rheological properties or changes in droplet sizes were observed over a period of one month. However, emulsions exhibited a creaming phenomenon wherein droplets rose through the matrix fluid under the influence of gravity [11]. Figure 18 shows the samples of emulsions left unstirred for several weeks. The following points should be noted from Figure 18: (1) the creaming of emulsions decreases with the increase in oil concentration, at any given nanoparticle concentration; (2) the creaming effect in emulsions decreases with the increase in nanoparticle concentration; and (3) the creaming effect is almost completely eliminated at high concentrations of oil and nanoparticles. Figure 19 shows the plots of percent creaming index (%*CI*) versus oil concentration for emulsions at different nanoparticle concentrations where %*CI* is defined as:

(3) $$\%CI=\left(H_{S}/H_{E}\right)\times 100$$

$H_{S}$ is the height of serum layer (layer free of oil droplets) at the bottom and $H_{E}$ is the height of the total emulsion sample. For a given nanoparticle concentration, the creaming index decreases rapidly with the increase in oil concentration. The creaming index also decreases rapidly with the increase in nanoparticle concentration at any given oil concentration. 

## 4. Conclusions

Based on the experimental work and data analysis, the following conclusions can be made:

- The O/W emulsions thickened with starch nanoparticles are generally Newtonian at low concentrations of oil. At high concentrations of oil, the emulsions become non-Newtonian shear-thinning.
- The flow behavior of O/W emulsions thickened by starch nanoparticles can be described adequately using the power-law model.
- The power-law consistency index increases with increases in oil and nanoparticle concentrations.
- The power-law flow behavior index decreases with increases in oil and nanoparticle concentrations, indicating that emulsions become more shear-thinning with increases in oil and nanoparticle concentrations.
- The average droplet size of emulsions generally decreases with an increase in the nanoparticle concentration of the matrix phase. The droplet size distribution also becomes narrower with an increase in nanoparticle concentration.
- The creaming index of emulsions decreases the increases in oil and nanoparticle concentrations.

## Figures and Tables

**Table 1:** Composition of emulsion-based food products [7].

Food Product	Dispersed Phase	Continuous Phase	Volume Fraction of Dispersed Phase
Milk	Oil droplets	Water	0.03 to 0.04
Mayonnaise	Oil droplets	Water	≥0.65
Butter	Water droplets	Oil and fat crystals	About 0.16
Margarine	Water droplets	Oil and fat crystals	0.16 to 0.50
Salad dressings	Oil droplets	Water	≥0.30

**Table 2:** Compositions of emulsions investigated in this study.

Nanoparticle Concentration of Matrix Phase (wt%)	Nanoparticle Concentration of Matrix Phase (vol%)	Oil Concentration of Emulsion (wt%)	Oil Concentration of Emulsion (vol%)
0	0	Seven concentrations: 10.09, 20.05, 30.088, 40.03, 50.11, 60.06, 65.06	Seven concentrations: 11.674, 22.8, 33.636, 44.011, 54.19, 63.91, 68.68
5.245	3.27	Seven concentrations: 9.977, 19.962, 29.94, 39.974, 50, 59.973, 65	Seven concentrations: 11.21, 22.127, 32.745, 43.14, 53.256, 63.058, 67.906
9.883	6.6	Seven concentrations: 10.083, 20.153, 30.136, 40.142, 50.18, 60.13, 65.13	Seven concentrations: 12.041, 23.554, 34.493, 45.014, 55.147, 64.80, 69.51
15.032	10.233	Seven concentrations: 10.045, 20.01, 29.996, 39.99, 49.96, 59.99, 64.996	Seven concentrations: 12.20, 23.73, 34.78, 45.33, 55.408, 65.108, 69.794
19.68	13.64	Seven concentrations: 9.999, 20.04, 30, 40.023, 50.058, 60.015, 65.022	Seven concentrations: 12.334, 24.094, 35.184, 45.802, 55.934, 65.526, 70.186
24.82	17.541	Seven concentrations: 10, 19.982, 29.977, 40.04, 49.98, 59.95, 64.97	Seven concentrations: 12.563, 24.39, 35.61, 46.313, 56.34, 65.915, 70.55

**Table 3:** Relevant dimensions of viscometers used in this study.

Device	Inner Cylinder Radius, *R_i_*	Outer Cylinder Radius, *R_o_*	Length of Inner Cylinder	Gap-Width
Fann 35A/SR-12	1.7245 cm	1.8415 cm	3.8 cm	0.117 cm
Haake Roto-visco RV 12 with MV I	2.004 cm	2.1 cm	6.0 cm	0.096 cm

**Table 4:** Sauter mean diameters of O/W emulsions thickened with starch nanoparticles. The oil concentration is fixed at approximately 65 wt%.

Approximate Nanoparticle Concentration (wt% NPS)	Sauter Mean Diameter (µm)
0	9.01
5	7.31
10	4.68
15	3.71
20	4.46
25	5.64

## Data Availability

The data presented in this study are available on request from corresponding author.
